# A Systematic Review of Lipid-Focused Cardiovascular Disease Research: Trends and Opportunities

**DOI:** 10.3390/cimb45120618

**Published:** 2023-12-09

**Authors:** Uchenna Alex Anyaegbunam, Piyush More, Jean-Fred Fontaine, Vincent ten Cate, Katrin Bauer, Ute Distler, Elisa Araldi, Laura Bindila, Philipp Wild, Miguel A. Andrade-Navarro

**Affiliations:** 1 Computational Biology and Data Mining Group (CBDM), Institute of Organismic and Molecular Evolution (iOME), Johannes Gutenberg University, 55122 Mainz, Germany; 2 Department of Pharmacology, University Medical Center Mainz, 55131 Mainz, Germany; 3 Central Institute for Decision Support Systems in Crop Protection (ZEPP), 55545 Bad Kreuznach, Germany; 4 Preventive Cardiology and Preventive Medicine, Department of Cardiology, University Medical Center of the Johannes Gutenberg University Mainz, Langenbeckstr. 1, 55131 Mainz, Germany; 5 Clinical Epidemiology and Systems Medicine, Center for Thrombosis and Hemostasis (CTH), University Medical Center, 55131 Mainz, Germany; 6 German Center for Cardiovascular Research (DZHK), Partner Site Rhine Main, University Medical Center of the Johannes Gutenberg University Mainz, 55131 Mainz, Germany; 7 Computational Systems Medicine, Center for Thrombosis and Hemostasis (CTH), 55131 Mainz, Germany; 8 Institute of Immunology, University Medical Center of the Johannes Gutenberg University Mainz, 55131 Mainz, Germany; 9 Research Centre for Immunotherapy (FZI), University Medical Center of the Johannes Gutenberg University Mainz, 55131 Mainz, Germany; 10 Institute of Physiological Chemistry, University Medical Center, 55131 Mainz, Germany

**Keywords:** lipids, cardiovascular diseases, lipoproteins, lipid-protein network, research trends

## Abstract

Lipids are important modifiers of protein function, particularly as parts of lipoproteins, which transport lipophilic substances and mediate cellular uptake of circulating lipids. As such, lipids are of particular interest as blood biological markers for cardiovascular disease (CVD) as well as for conditions linked to CVD such as atherosclerosis, diabetes mellitus, obesity and dietary states. Notably, lipid research is particularly well developed in the context of CVD because of the relevance and multiple causes and risk factors of CVD. The advent of methods for high-throughput screening of biological molecules has recently resulted in the generation of lipidomic profiles that allow monitoring of lipid compositions in biological samples in an untargeted manner. These and other earlier advances in biomedical research have shaped the knowledge we have about lipids in CVD. To evaluate the knowledge acquired on the multiple biological functions of lipids in CVD and the trends in their research, we collected a dataset of references from the PubMed database of biomedical literature focused on plasma lipids and CVD in human and mouse. Using annotations from these records, we were able to categorize significant associations between lipids and particular types of research approaches, distinguish non-biological lipids used as markers, identify differential research between human and mouse models, and detect the increasingly mechanistic nature of the results in this field. Using known associations between lipids and proteins that metabolize or transport them, we constructed a comprehensive lipid–protein network, which we used to highlight proteins strongly connected to lipids found in the CVD-lipid literature. Our approach points to a series of proteins for which lipid-focused research would bring insights into CVD, including Prostaglandin G/H synthase 2 (PTGS2, a.k.a. COX2) and Acylglycerol kinase (AGK). In this review, we summarize our findings, putting them in a historical perspective of the evolution of lipid research in CVD.

## 1. Introduction 

Lipids are relevant molecules in a number of biological processes, particularly as part of membranes, modifying and regulating their properties, and as signalling molecules or more passively as energy storage in the adipose tissue. Notably, in the circulating blood, lipids and their associated proteins can be quantified and are valuable as indicators of various diseases [1]. For instance, in the realm of cardiovascular disease (CVD)—a prominent cause of global mortality influenced by environmental factors, such as diet, smoking and alcohol consumption [2]—lipid biomarkers such as low-density lipoprotein (LDL) cholesterol and high-density lipoprotein (HDL) cholesterol are pivotal. LDL cholesterol, deemed ‘bad’ cholesterol, is implicated in atherogenesis, whereas HDL cholesterol, the ‘good’ counterpart, is associated with cardiovascular protection. Additionally, triglycerides, when elevated alongside low HDL levels, escalate CVD risk [3]. Beyond lipids themselves, apolipoproteins like Apolipoprotein B (ApoB) in LDL, and Apolipoprotein A1 (ApoA1) in HDL, are instrumental in gauging CVD risk [4]. These molecular markers are vital for appraising and strategizing CVD risk management.

The particularly complex interplay between environment and phenotype in CVD affects the research of the cause–effect relations between lipids and CVD [5]. This is well illustrated by the long-standing debate on the influence of diet in the physiological balance of lipids and its effect on CVD. An early editorial article from George Mann in 1977 exposed the lack of evidence for the efficacy of diet therapy for the treatment of coronary heart disease [6]. The levels of cholesterol in blood are associated with CVD and atherosclerosis, but this is not so much influenced by diet and is due more to insufficient metabolism [7]. Research into the products of oxidation of cholesterol and how these affect enzymes that process lipids in feedback loops followed [8]. The belief that a diet high in saturated fat results in high levels of blood cholesterol, and that this causes atherosclerosis and CVD, was challenged, and exercise was presented as a better measure to prevent heart disease than diet [9]. To this day, we are far from understanding the effects of diet, stress and exercise on CVD [10].

In recent decades we have witnessed the development of databases and high-throughput techniques, with the expectation that this would enhance our mechanistic insights into the role of lipids in CVD. However, while the advent of sequencing technologies, transcriptomics and proteomics has provided a wealth of molecular information and cell regulatory data, lipidomics, the identification of thousands of lipids from a sample, typically by mass spectrometry, suffers from particular difficulties in the precise identification of lipidic molecules (e.g., some isoforms cannot be distinguished) [11], which necessitated an updated nomenclature. We wondered if we could detect an evolution of the narrative surrounding lipids in CVD research in the literature towards molecular pathomechanisms while at the same time identifying possible knowledge gaps that could constitute opportunities for advancing the field. 

To approach an analysis of topics in the literature, we took advantage of the annotations of records in the PubMed database of biomedical literature [12] with Medical Subject Heading (MeSH) terms. MeSH terms were started in 1963 [13] to summarize the content of biomedical publications. They facilitate keyword searches, for example by grouping multiple synonyms like tumour or tumor, cancer and malignancy under the umbrella MeSH term “Neoplasms”. In addition, MeSH terms have been used for the identification of trends in research [14,15]. Qualifiers narrow the application of given MeSH terms (https://www.nlm.nih.gov/mesh/subhierarchy.html - accessed on 5 December 2023). For example, the MeSH term “Aging” currently has 13 allowed qualifiers including “genetics”, “radiation effects” and “physiology”. The MeSH term annotations for lipids and diseases are of high quality and allowed finding associations of lipids with human diseases from the literature [16]. Here, we exploit these annotations of lipids, diseases and qualifiers to systematically review the different approaches used to research lipids in CVD and the trends in this research.

Finally, since the dysregulation of the balance of lipids could be detected by lipidomics in human blood plasma, we study the network of plasma proteins and the lipids they metabolize and transport in the context of CVD. Evaluating these protein–lipid connections across multiple studies reveals potential targets of interest in lipid-focused CVD research. Our findings could open doors to new avenues of research in CVD and lipids.

## 2. Methods

A systematic review of PubMed articles related to lipids and cardiovascular diseases was performed, adhering to the most recent Preferred Reporting Items for Systematic Reviews and Meta-Analyses (PRISMA) guidelines. The review process was registered with the international prospective register of systematic reviews (PROSPERO) under the registration number CRD42023474223.

### 2.1. Selection of PubMed Records and Data Visualization

The list of lipids and corresponding PubChem CIDs were obtained from LIPID MAPS (March 2023; [17]). We then queried PubMed (March 2023) to obtain a dataset of PubMed records associated with lipid PubChem CIDs (see Flow diagram). These records were further queried as: “(Humans OR Mice) AND (Plasma OR Heart OR Myocardium)” and containing at least one MeSH term for CVD (category C, child of MeSH term “Cardiovascular Diseases”) and one MeSH term for a lipid (category D, child of MeSH term “Lipids”; Supplementary Table S1). The result was further selected and annotated for their association with human or mouse (from the MeSH terms category B “Humans” and “Mice”), and association with plasma, heart or myocardium (from the MeSH terms category A “Plasma”, “Heart”, “Myocardium”).

Heat maps of term use were carried out using the Seaborn Python data visualization library.

### 2.2. Association between Terms

We evaluated the co-occurrence of Lipids and CVD MeSH terms (Supplementary Table S2), Lipids and MeSH qualifiers (Supplementary Table S3) and CVDs and MeSH qualifiers (Supplementary Table S4). Significance of all pairwise co-occurrences was evaluated using the Fisher’s exact test. Top associations are shown in Table 1. There, Fabry disease has been implicated to be highly associated with some lipids. Although Fabry disease is a lysosomal storage disease, cardiovascular complications are an important part of its pathogenesis. Indeed, the Medical Subject Headings (MeSH) hierarchy includes conditions like Fabry disease, mitochondrial diseases such as MELAS (Mitochondrial Encephalomyopathy, Lactic Acidosis, and Stroke-like episodes), and genetic syndromes such as Noonan syndrome under the category of cardiovascular diseases because these disorders often have cardiovascular manifestations as part of their clinical presentation. Including these diseases under the cardiovascular category allows for a more integrated approach to research and treatment, acknowledging the significant impact these conditions have on the cardiovascular system even if they are not primarily heart diseases. This categorization reflects the understanding that the cardiovascular system does not operate in isolation but is affected by and interacts with other systems within the body.

### 2.3. Construction of a Lipid–Protein Network

To build a network relating proteins and the lipids they metabolize or transport, with chances of being used in studies of metabolomics, we started with a list of lipids previously assessed as reliably quantifiable in human plasma [18]. We extracted the proteins associated with these lipids from the SwissLipids database (March 2023; [19]). Gene Ontology enrichment analysis of these proteins revealed their involvement in metabolic processes. We subsequently queried these proteins in the SwissLipids database to extract their metabolic reaction equations with lipids. A total of 451 lipids and 92 proteins were identified. Using this information, we constructed a lipid–protein network using iGraph (version 1.3.5) in the R language. To simplify the graph, duplicate interactions were removed.

## 3. Results and Discussion

**Figure 1:**
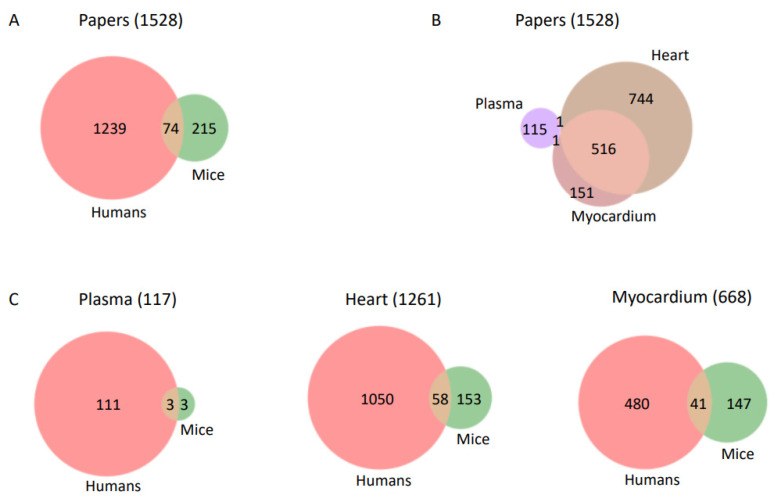
Distribution of categories in papers associated with lipids and CVD. (**A**) Overlap between papers associated with human and mouse. (**B**) Overlap between papers associated with plasma, heart and myocardium. Only one paper was associated with plasma and heart, and another one with plasma and myocardium. (**C**) Overlap between papers associated with human and mouse for each of the three tissue/organ categories: plasma, heart, myocardium. Circles are not to scale for convenience of representation.

To acquire knowledge about the topics and trends of research in the field of lipids and CVD, we produced a targeted selection of records from PubMed. For this, we focused on content annotated as dealing both with lipids and cardiovascular diseases and associated with species human or mouse (which is often used as CVD model; e.g., [20,21,22,23,24]). To further focus on physiological research and blood biological markers (biomarkers), we selected records also annotated as investigating the following tissues: plasma, heart or myocardium (see Methods for details). This approach resulted in 1,528 records associated with both lipids and CVD and also with at least one of the three tissues of interest (Figure 1; Supplementary Table S1). Note that while there were 199 records for articles associating lipids and CVD in human and mouse published between 2021 and 2023, none of them were yet annotated with any of the three tissue terms (as of March 2023) and hence they did not end up in our database.

We found almost five times as many articles investigating humans as mice (Figure 1A). Many more articles were obtained for heart (n = 1261) and myocardium (n = 668) than for plasma (n = 117); most articles dealing with the myocardium also discuss the heart because the latter term includes the former, while overlap with plasma was minimal (Figure 1B). Regarding the three tissues and the two species, most plasma research was focused on humans (highlighting the use of human blood lipid levels as CVD biomarkers); research in mice was more biased towards myocardium than heart, but human research dominated both (Figure 1C).

### 3.1. Temporal Trends in Lipid-Focused CVD Research

**Figure 2:**
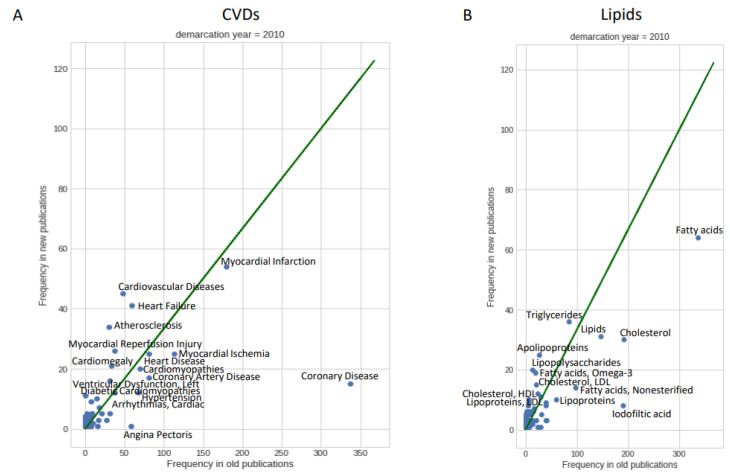
Differences of frequency of MeSH CVD and lipid terms in new versus old publications. (**A**) For CVD. (**B**) For lipids. Old publications are from before 2010 and new publications are from the year 2010 and after. The diagonal represents the ratio of new versus old publications (356/1172 = 0.30).

We studied the temporal trends in the set of selected publications according to their annotations (MeSH disease and lipid terms). To find recent shifts in focus of research, we divided the sample into articles published before 2010 (old publications; 1172 records) and a smaller number (but large enough to allow us to derive meaningful trends) of those published in 2010 and afterwards (new publications; 356 records). We then compared disease and lipid term usage in both sets (Figure 2).

Regarding diseases, we observe an increase over time in disease terms such as atherosclerosis, heart failure, myocardial reperfusion injury and diabetic cardiomyopathies, while terms like myocardial ischemia, hypertension, arrhythmia, cardiomyopathies, coronary artery disease and (most prominently) coronary disease are less frequent (dots above and below the diagonal in Figure 2A, respectively). We interpret these results as indicating a shift of lipid-focused CVD research from the effects of disease (hypertension, arrhythmia, myocardial ischemia) to the diseases themselves.

Regarding lipids, apolipoproteins and triglycerides are on the rise, while general terms like lipids, cholesterol and fatty acids are less used in the more recent literature (Figure 2B). These trends align with the increasing general specialization in biomedical sciences. The case of iodofiltic acid is related to its (decreasing) usage as imaging marker (see more details below). 

**Figure 3:**
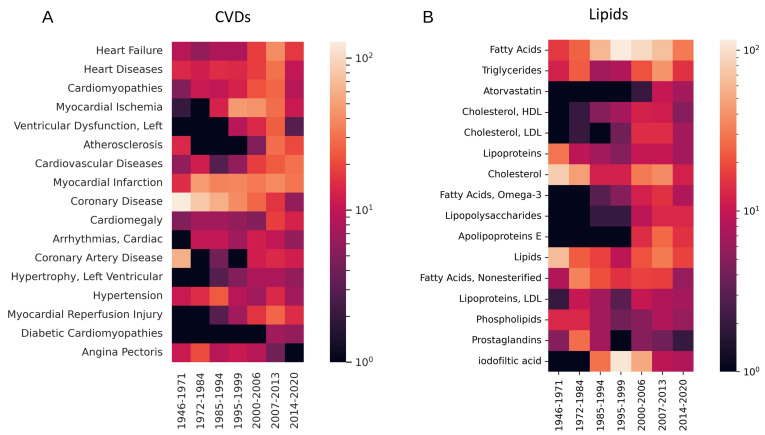
Evolution of usage of frequent MeSH CVD and lipid terms. (**A**) For CVD. (**B**) For lipids. The values represent numbers of articles using the term.

To identify concepts with a peak time, we carried out a more detailed time analysis dividing the set of articles into seven periods that contained similar numbers of articles (approximately 200 each; Figure 3). This analysis shows again that coronary disease is a topic falling into disuse (since the 1970s) and indicates that angina pectoris was most studied around 1980, hypertension around 1990, and myocardial ischemia around 2000, confirming the shift in research from symptoms to diseases. Cardiomegaly and diabetic cardiomyopathies look like topics trending in recent years (Figure 3A). For lipids, the peaks for iodofiltic acid and fatty acids in 1995–1999 are very strong (Figure 3B). Minima (indicating comeback of topics) do not seem to be common among these very frequent terms. Care must be taken when interpreting these results, as they may reflect variations in the annotation procedure over time, but as indicated in the introduction, the quality of MeSH term annotations is high, since it allowed us to obtain useful information in several previous applications of data and text mining for the extraction of associations between biological molecules and diseases [25,26].

**Figure 4:**
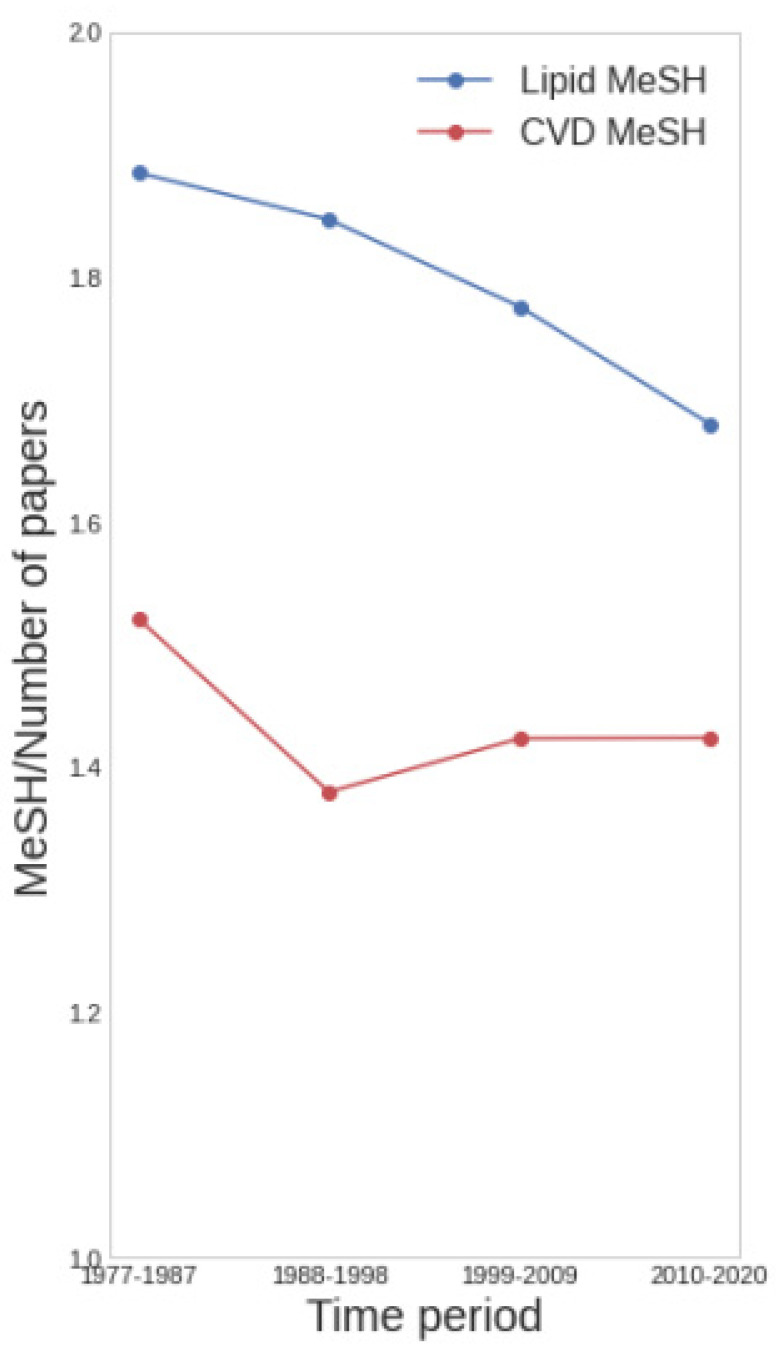
Average number of MeSH CVD and lipid terms per paper versus time. CVD MeSH terms per paper (red) and Lipid MeSH terms per paper (blue) in four different time periods.

To address possible trends in the multifactorial character of lipid-focused CVD research, we used the number of topics per record as a measure of the complexity of the research. In general, more than one Lipid MeSH term and more than one CVD MeSH term were found per paper (average of 1.70 and 1.47, respectively). To analyse temporal trends, we divided the dataset into four time periods (with about 400 records each) and found a fairly constant ratio of around 1.4 CVD terms (Figure 4). The values for lipids did not change a lot in different years although we could observe a small but sustained decreasing trend (Figure 4). This could indicate increasing specialization in the field with respect to the lipids under analysis, but as mentioned above, it could also be due to trends in the annotation procedure.

### 3.2. Qualifiers of Lipid Research in CVD

We analysed the association between the two MeSH term types, Lipids and CVDs, and also between each type and MeSH qualifiers, in our set of selected literature to obtain a better understanding of the points of view of research connecting these terms (Table 1 and Supplementary Tables S2–S4). These associations were computed by their co-occurrence in the annotations of the same records (See Methods for details).

In the associations between Lipids and CVD terms (Table 1; Supplementary Table S2), we can find known associations, such as apolipoprotein E (ApoE) in atherosclerosis, ceramides in Fabry Disease, cholesterol and lipoproteins in arteriosclerosis, and so on. While these associations are not too surprising (and reproduce previous work [16]), they support our methodology in evaluating associations between different types of terms, which we will use next with MeSH qualifiers.

Regarding associations between lipids and MeSH qualifiers (Table 1; Supplementary Table S3), the strongest association found reflected the fact that iodofiltic acid is used in diagnostic imaging. Lipids and cholesterol are studied in the context of blood. Fish oils and omega-3 are associated with disease prevention, suggesting a low connection to molecular biology in the study of these lipids. A very different status of research is shown for ApoE, which is associated both with genetics and deficiency, suggestive of studies of deeper mechanistic insight.

In the list of associations between CVDs and MeSH qualifiers, we can see that arrhythmias are chemically induced (in murine models) (Table 1; Supplementary Table S4). Lipids in sudden cardiac death and myocardial reperfusion injury are studied for their prevention (linked to the lipids mentioned above). Other interesting specific aspects of lipids in CVD research emerge, such as the immunological aspect in myocarditis research, and cytology in cardiomegaly.

### 3.3. Research of Lipids in CVD and the Lipid-Protein Network

**Figure 5:**
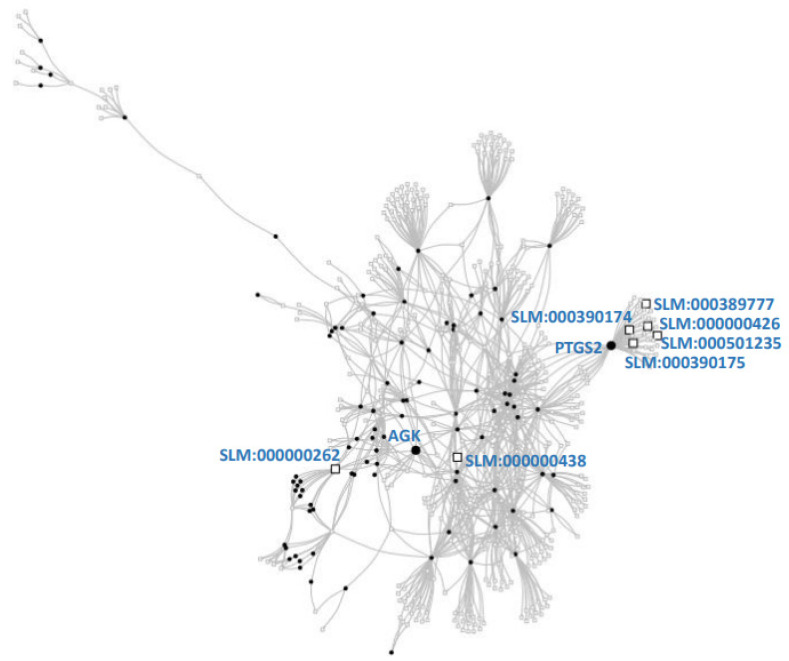
Protein–lipid network and top two proteins selected. The network represents proteins (squares) connected to lipids (dots) that they metabolize or transport. Literature lipids (large squares; Supplementary Table S6) were used to score proteins connected to them: prostaglandin G/H synthase 2 (PTGS2; UniProt:P35354) and acylglycerol kinase (AGK; UniProt:Q53H12) were the two most connected proteins (large dots). See Table 2 for other highly scored proteins (the full list is available as Supplementary Table S7). See text and Methods for details.

We observed that 111 of our set of 1528 papers were associated with a human protein (Supplementary Table S1). To find out if this subset addresses proteins relevant for lipid research according to known molecular relations between proteins and highly investigated lipids, we first constructed a network of proteins and lipids using information about the proteins that metabolize or transport those lipids, as indicated in databases (see Methods for details). Additionally, this network forms a resource to identify novel proteins that may be of importance for lipid research in CVD. The resulting network contains 92 proteins and 451 lipids connected via 967 edges that can indicate metabolism or transport (Figure 5; Supplementary Table S5).

Next, we applied a simple scoring method where we first identify the lipids in the network that were found in our literature set, and then we score all proteins in the network so that each protein receives 1/n points from each lipid found in our literature set situated at n metabolic steps from the protein (the more metabolically distant a protein is from the literature lipids, the lower its score). 

Only two lipid literature terms corresponded to lipids in the network: sphingosine 1-phosphate and prostaglandin H2. Another two literature lipid terms (1,2-diacylglycerol, prostaglandins) corresponded to categories encompassing another five lipids in the network (Supplementary Table S6; large squares in Figure 5). Using these seven lipids in the network, we ranked all proteins (Supplementary Table S7; top two marked by large dots in Figure 5).

The top-ranked protein was prostaglandin G/H synthase 2 (PTGS2; UniProt: P35354), a.k.a. Cyclooxygenase-2 (COX2). This protein did not appear in the literature selected for CVD and lipids. Indeed, PTGS2 has been studied in the context of coronary artery atherosclerosis [27] and heart failure in a rat model [28], and its cardioprotective role has been reviewed [29,30]. While the isozyme PSTG1 (a.k.a. COX1) is constitutive, PTGS2 is inducible, probably responsible for lipid biosynthesis in inflammation. These publications were absent from our selection due to our strict selection criteria of lipid-focused research. The second-ranked protein (also absent from our selection of lipid-focused CVD papers) is the mitochondrial acylglycerol kinase (AGK; UniProt: Q53H12). Mutations and splice variants of this protein cause Sengers syndrome [31,32,33], which includes cardiomyopathy, among other symptoms. This could be due to citrate synthase precipitates [34]. A few other high-scoring candidate proteins are detailed in Table 2, and a full list is available as FLO Supplementary Table S7.

**Table 2:** Top proteins ranked according to their association with CVD-literature-selected lipids. Full table is available as Supplementary Table S6.

UniProt	Score	Protein Name	Gene ID	CVD References
P35354	5.8	Prostaglandin G/H synthase 2	PTGS2	[22,23,24,25]
Q53H12	4.0	Acylglycerol kinase, mitochondrial	AGK	[26,27,28,29]
Q9NUN7	3.5	Alkaline ceramidase 3	ACER3	
Q9NWW9	3.5	Phospholipase A and acyltransferase 2	PLAAT2	
P53816	3.5	Phospholipase A and acyltransferase 3	PLAAT3	[32,33]
Q9HDD0	3.5	Phospholipase A and acyltransferase 1	PLAAT1	
Q9UL19	3.5	Phospholipase A and acyltransferase 4	PLAAT4	

Proteins with lower scores (less connected to literature-selected lipids) but lacking CVD-related literature could constitute potentially interesting targets for novel lines of lipid-focused CVD-related research. The case of alkaline ceramidase 3 (ACER3) is suggestive because its expression has been found to be regulated by myriocin, an inhibitor of the ceramide biosynthesis pathway in a rat model of cerebral stroke [35]; myriocin is used to treat myocardial ischemia/reperfusion injury. This protein, among other ceramidases, has been discussed as a possible target to treat diseases of altered lipid homeostasis [36]. 

We also note the few members of the set of phospholipase A and acyltransferase paralogs (PLAAT1, 2 and 4) that lack a CVD-related bibliography (see Table 2), while PLAAT3 (a.k.a. PLA2G16) has been found in a GWAS study associated with Chagas cardiomyopathy [37] and was the most downregulated gene in calf muscle of patients diagnosed with peripheral artery disease (PAD) [38]. 

Together, these results suggest that our ranked list points to proteins for which lipid-focused research could help in understanding the involvement of those proteins in CVD.

Finally, we checked whether the proteins in our lipid–protein network are mentioned in our literature subset of 111 papers associated with human proteins and found only one: the microsomal triglyceride transfer (MTP) protein large subunit (UniProt: P55157), which transfers lipids into apolipoprotein B-containing lipoproteins. The corresponding paper reports that a variant of the MTP gene’s promoter (MTP-493G/T, a G to T polymorphism 493 base pairs upstream from the start of transcription) increases the risk of coronary disease and produces a reduction in plasma LDL cholesterol levels [39] (Supplementary Table S1). In our network, this protein is connected to four lipid terms: 1,2-diacyl-sn-glycero-3-phosphocholine (SLM:000000261), 1,2-diacyl-sn-glycero-3-phosphoethanolamine (SLM:000000239), cholesterol esters (SLM:000000470) and triacyl-sn-glycerol (SLM:000000353). While this paper assessed the levels of plasma cholesterol, triglycerides, LDL cholesterol and HDL cholesterol, our simple analysis would suggest that testing the levels of 1,2-diacyl-sn-glycero-3-phosphocholine and 1,2-diacyl-sn-glycero-3-phosphoethanolamine could better reveal the dysfunction of the gene.

## 4. Conclusions

Considering the involvement of lipid metabolism in CVD and also the improvement of techniques for exploring molecular networks and genetics over the years, we selected literature associated with lipids and CVD to identify trends in lipid-focused research of CVD and to evaluate new possibilities of research relevant in advancing our understanding of the roles of lipids in the molecular mechanisms of CVD.

To focus our study on lipids in circulating blood and on the heart, we required our literature selection to mention the tissues, plasma, heart and myocardium. Considering the extensive use of animal models in CVD, in addition to the literature that considers humans, we added research in mice. We were surprised to find a mere 16% increase in our dataset, reflecting the homocentric focus of the field (Figure 1A). It is true that rodents (mice and rats) are not ideal CVD models due to molecular differences in cardiac physiology, such as the expression of alpha-myosin heavy chain (instead of beta-myosin heavy chain in large mammals) in the ventricles and differences in lipid metabolism [40]. Nevertheless, a significant increase in myocardium-related research (28%) was obtained, not so much for heart (14%) (Figure 1C). Addition of other animal models closer in physiology to humans, such as pigs, might provide additional insights.

Comparison of older (before 2010) and more recent (in 2010 or after) bibliographic topics indicated a shift of lipid-focused CVD research from symptoms to diseases, save for heart failure (Figure 2A). This reflects an increase in the power to study mechanisms instead of reporting observations. Regarding lipids in CVD, there is a lower use of terms like cholesterol or fatty acids, in favour of more specific terms such as Omega-3, HLD Cholesterol and LDL Cholesterol (Figure 2B). A more detailed analysis confirmed these trends (Figure 3). Taking the number of terms analysed per paper as an indication of the multifactorial aspects of lipid-focused CVD research, a fairly constant ratio of around 1.4 CVDs mentioned per paper suggests that the field does deal with the multifactorial aspects of CVD to some degree; the slight but steady decrease in lipid terms used per paper from 1.9 around 1980 to 1.7 in the most recent literature is similarly suggestive of a trend towards more focused molecular research (Figure 4). The constant rate for CVD terms mentioned per paper would suggest that researchers could not disentangle the different aspects of CVD or that new related diseases have come into focus. The emergence of new disease topics at different time points (Figure 3) supports the latter possibility.

As more biological lipids are investigated and the capabilities to measure them increase, the evidence that dysregulation of lipids in circulating blood (dyslipidaemia) can lead to disease increases: this makes lipids appealing molecular biomarkers. Biomarkers are generally used for diagnostic or prognostic purposes [41]. As a result, the increased focus of the field on molecular mechanisms can also be observed in the increment of records annotated with the MeSH term “Biomarkers” in recent publications (Supplementary Table S1). In our literature selection, “Biomarkers” is found in 15 records published before 2010 (1.2% of 1172 in total) and in 22 published in or after 2010 (6.2% of 356 records). A molecular trend can also be observed if we count the number of records associated with a human protein: 58 in papers published before 2010 (5.0% of records) and 63 in the papers published in or after 2010 (17.7% of records) (Supplementary Table S1), indicating that the molecular interactions of lipids with proteins are receiving increasing attention.

Following this idea, we ranked proteins associated with lipid metabolism and transport based on the lipids mentioned in our literature dataset. Our ranked list contained some well-known proteins at the top, but also suggested some potentially new targets for lipid-focused CVD research (Table 2). We provide these datasets and analyses as a resource to increase awareness in the field of clinical lipid research in CVD. 

In this work, we have demonstrated how to generate a network of lipids and metabolically related proteins, which we used to evaluate proteins by a list of lipids selected from the literature and vice versa using a simple method. The future availability of new studies comparing lipidomic and proteomic profiles should allow us to optimize and extend such methods.

The advent of extended cohort-based research and widespread use of lipidomics and proteomics, in addition to transcriptomics, should facilitate network approaches like the one taken here. These approaches will improve the selection of lipids and proteins to be used as markers with increased specificity and mechanistic knowledge and should have an impact in the prognosis and therapy of CVD.

## Figures and Tables

**Table 1:** Top 10 associations between specific lipids and cardiovascular diseases, along with their research prevalence and statistical significance. It shows strong links between apolipoproteins E and atherosclerosis, and lipids like trihexosylceramides with Fabry Disease. Omega-3 fatty acids are notably associated with cardiac events. The table also connects MeSH qualifiers with lipids and CVDs, indicating frequent research intersections, such as diagnostic imaging with iodofiltic acid and fatty acids. Extended data are available in Supplementary Tables S2–S4.

Lipid	CVD	#Papers Lipid	#Papers CVD	#Papers Lipid & CVD	*p*-Value
Apolipoproteins E	Atherosclerosis	52	64	24	4.06 × 10^−14^
Trihexosylceramides	Fabry Disease	7	11	7	2.45 × 10^−11^
Globotriaosylceramide	Fabry Disease	6	11	6	5.76 × 10^−10^
Fatty Acids, Omega-3	Death, Sudden, Cardiac	38	25	11	2.32 × 10^−9^
Cholesterol	Arteriosclerosis	222	84	37	6.26 × 10^−7^
Fatty Acids, Omega-3	Arrhythmias, Cardiac	38	49	10	4.1 × 10^−6^
Fish Oils	Death, Sudden, Cardiac	21	25	6	7.63 × 10^−6^
Fats	Coronary Artery Disease	31	98	12	1 × 10^−5^
Glycolipids	Fabry Disease	3	11	3	1.17 × 10^−5^
Lipoproteins	Arteriosclerosis	70	84	16	2.01 × 10^−5^
**Lipid**	**MeSH Qualifier**	**#Papers Lipid**	**#Papers MeSH q**	**#Papers Lipid & MeSH q**	***p*** **-Value**
Iodofiltic acid	diagnostic imaging	617	819	195	4.59 × 10^−27^
Fatty Acids	diagnostic imaging	1552	819	273	5.5 × 10^−20^
Apolipoproteins E	deficiency	352	142	29	1.45 × 10^−17^
Fatty Acids, Omega-3	prevention & control	230	301	30	5.18 × 10^−13^
Cholesterol	blood	1027	988	181	9.54 × 10^−13^
Apolipoproteins E	genetics	352	405	38	1.47 × 10^−11^
Iodofiltic acid	pharmacokinetics	617	134	38	5.94 × 10^−10^
Lipids	blood	785	988	137	2.36 × 10^−9^
Fish Oils	prevention & control	143	301	17	2.84 × 10^−8^
Lipopolysaccharides	toxicity	272	84	12	7.93 × 10^−8^
**CVD**	**MeSH Qualifier**	**#Papers CVD**	**#Papers MeSH q**	**#Papers Lipid & MeSH q**	***p*** **-Value**
Chagas Cardiomyopathy	parasitology	6	6	6	4.46 × 10^−11^
Death, Sudden, Cardiac	prevention & control	25	159	16	6.74 × 10^−7^
Myocarditis	immunology	13	45	7	1.47 × 10^−6^
Atherosclerosis	deficiency	64	68	15	3.15 × 10^−6^
Cardiomegaly	cytology	54	61	12	1.60 × 10^−5^
Myocardial Reperfusion Injury	prevention & control	63	159	22	1.86 × 10^−5^
Myocardial Reperfusion Injury	pharmacology	63	368	38	2.87 × 10^−5^
Heart Neoplasms	secondary	3	2	2	5.08 × 10^−5^
Cardiomyopathy, Hypertrophic	diagnostic imaging	30	432	26	6.13 × 10^−5^
Arrhythmias, Cardiac	chemically induced	49	92	13	6.58 × 10^−5^

## Data Availability

All data generated or analysed during this study are included in this published article [and its supplementary information files].

## Supplementary Materials

The following supporting information can be downloaded at: https://www.mdpi.com/article/10.3390/cimb45120618/s1, Supplementary Table S1. Selection of 1528 records from PubMed associated with lipids and CVD. Columns indicate PMID, publication year, all MeSH terms, chemical MeSH ids and terms, CVD MeSH ids and terms, lipid MeSH names, CVD MeSH terms, CVD MeSH names, MeSH qualifiers, then whether papers are associated with humans, mice, plasma, heart, myocardium or biomarkers (0 and 1 indicate no association or association, respectively), and the associated proteins (UniProtID; NA means none available). Supplementary Table S2. Associations of lipids and CVDs. Columns indicate lipid name, CVD, number of papers mentioning the lipid, the CVD, or both, and *p*-value of association (Fisher’s exact test). Supplementary Table S3. Associations of lipids and MeSH qualifiers. Columns indicate lipid name, MeSH qualifier, number of papers mentioning the lipid, the MeSH qualifier, or both, and *p*-value of association (Fisher’s exact test). MeSH q: MeSH qualifiers. Supplementary Table S4. Associations of CVDs and MeSH qualifiers. Columns indicate CVD name, MeSH qualifier, number of papers mentioning the CVD, the MeSH qualifier, or both, and *p*-value of association (Fisher’s exact test). MeSH q: MeSH qualifiers. Supplementary Table S5. Protein–lipid network. Connected pairs of lipids and proteins (UniProt IDs) are indicated. Supplementary Table S6. Lipids from the CVD literature. The comment column indicates if the term was directly found in the CVD literature (two cases) of if the class was found instead (five cases). Supplementary Table S7. Ranked proteins. Proteins in the protein–lipid network were ranked according to a list of seven lipids mentioned in the CVD bibliography (Supplementary Table S6). Top ones are indicated with additional annotations in Table 2.
